# Circulating Non-Esterified Fatty Acids as Biomarkers for Fat Content and Composition in Pigs

**DOI:** 10.3390/ani11020386

**Published:** 2021-02-03

**Authors:** Marc Tor, Francesca Vilaró, Roger Ros-Freixedes, Javier Álvarez-Rodríguez, Lluís Bosch, Sofia Gol, Ramona N. Pena, Josep Reixach, Joan Estany

**Affiliations:** 1 Agrotecnio Center, Departament de Ciència Animal, Universitat de Lleida, 25198 Lleida, Spain; roger.ros@udl.cat (R.R.-F.); javier.alvarez@udl.cat (J.Á.-R.); romi.pena@udl.cat (R.N.P.); joan.estany@udl.cat (J.E.); 2 Scientific-Technical Services DATCEM, Universitat de Lleida, 25198 Lleida, Spain; francesca.vilaro@udl.cat; 3 Departament d’Enginyeria Química, Agrària i Tecnologia Agroalimentària, Universitat de Girona, 17071 Girona, Spain; lluis.bosch@udg.edu; 4 Selección Batallé S.A., Av. Segadors s/n, 17421 Riudarenes, Spain; sgol@batalle.com (S.G.); jreixach@batalle.com (J.R.)

**Keywords:** non-esterified fatty acids, intramuscular fat, oleic acid, fatty acid composition, stearoyl-CoA desaturase, leptin receptor, meat quality

## Abstract

**Simple Summary:**

Circulating non-esterified fatty acids (NEFA) may be valuable as biomarkers for intramuscular fat content and fatty acid composition, as well as for other meat quality traits, in finishing heavy Duroc pigs. However, circulating NEFA composition may be affected by other factors such as age, fasting duration, and genetic variants related with adipogenesis and fatty acid metabolism pathways (e.g., *SCD* and *LEPR*). This study revealed that the circulating NEFA composition, especially the oleic acid content, reflects the metabolic status of an animal at a given time but has limited value as biomarker of intramuscular fat content and fatty acid composition.

**Abstract:**

Circulating non-esterified fatty acids (NEFA) can reflect the composition of dietary fat or adipose tissues depending on the fasting conditions. Therefore, circulating NEFA may be valuable as biomarkers for meat quality traits, such as intramuscular fat content and fatty acid composition in finishing pigs. Genetic variants that regulate lipid metabolism can also modulate the circulating NEFA. We conducted an experiment with 150 heavy Duroc pigs to evaluate fluctuations in the circulating NEFA composition due to age, fasting duration and two genetic polymorphisms, one in the leptin receptor (*LEPR*; rs709596309) and one in the stearoyl-CoA desaturase (*SCD*; rs80912566) gene. Circulating NEFA were more saturated and less monounsaturated than the subcutaneous and intramuscular adipose tissues. Absolute circulating NEFA content was more influenced by fasting duration than age. The *SCD* polymorphism did not impact NEFA content or composition. The *LEPR* polymorphism affected the content but not the fatty acid composition. Circulating oleic acid NEFA content after a short fasting was positively correlated with intramuscular fat content and, after a long fasting, with intramuscular oleic acid content. We conclude that circulating NEFA reflect environmental and genetic metabolic changes but are of limited value as biomarkers for intramuscular fat content and fatty acid composition.

## 1. Introduction

Fat content and composition of pork has a decisive influence on its organoleptic, nutritional, and technological quality, either as fresh meat or elaborated products [1]. For example, in the production of high-quality dry cured products, for which high intramuscular fat contents are typically appreciated, enhanced pork quality can be pursued by favoring high oleic acid content rather than polyunsaturated fatty acids content to reduce the risk of rancidity during long curing periods. In this process it is necessary to have tools to understand and monitor the fat deposition processes that assist in animal husbandry decisions. Circulating non-esterified fatty acids (NEFA) could be a useful tool if they are validated as biomarkers for intramuscular fat content and fatty acid composition.

Circulating NEFA are key lipid crossroad metabolites that partake in the physiological mechanisms behind fat deposition. In post-prandial states, lipoprotein lipase catalyzes the hydrolysis of the triglyceride core of circulating chylomicrons and the released NEFA become available as substrate for triacylglycerol synthesis in the adipocyte cells. Thus, the post-prandial circulating NEFA composition closely reflects the composition of ingested triacylglycerols [2]. In contrast, in post-absorptive and fasting states, circulating NEFA are expected to mimic the adipose tissue fatty acid composition, because the adipose depots are the source from which energy reserves are mobilized to organs and tissues. In humans, NEFA dynamics have been modeled as a tool for the study of metabolic diseases [3] and NEFA have even been proposed as a useful surrogate biomarker for adipose tissue composition [4]. In pigs, the adipose tissue has been thoroughly studied because of its influence on the quality of pork, but there is scarce information available on circulating NEFA. Unlike in other species, in pigs most of the endogenous fatty acid synthesis takes place in the adipose tissue [5]. However, not all pigs have the same genetic predisposition to accumulate fat and thus, the circulating NEFA composition in the bloodstream is also likely to be influenced by their genetics. In particular, two genetic polymorphisms, one in the stearoyl-CoA desaturase (*SCD*) and one in the leptin receptor (*LEPR*) gene, are known to exert a major impact on the fat content and fatty acid composition [6,7]. The determination of NEFA in the bloodstream of pigs carrying distinct *LEPR* and *SCD* genotypes subjected to different fasting durations can contribute to characterize the dynamics of adipose tissue growth for fattening management. Nowadays, the analytical determination of circulating NEFA is easy and fast using ultra-performance liquid chromatography (UPLC) coupled to mass spectrometry (MS) for multiple reaction monitoring (UPLC-MRM), which avoid some classic sample preparation steps. These analytical methods have been proved to be more effective than traditional gas-liquid chromatography-based methods [8]. The aim of the present study was to: (i) characterize the circulating NEFA composition in growing Duroc pigs compared to adipose tissue and muscle, (ii) study the effect of age, fasting duration, and *SCD* and *LEPR* genotypes on the circulating NEFA content and composition, and (iii) assess the potential use of the NEFA content as biomarker for intramuscular fat content and fatty acid composition.

## 2. Materials and Methods

### 2.1. Animals and Sample Collection

Pigs used in this study were obtained from a purebred Duroc line (Selección Batallé, Riudarenes, Girona, Spain). A total of 150 barrows were raised in two batches (70 and 80 animals) spaced over time. At approximately 75 d of age, the pigs were moved to the fattening units housed at 1m^2^/pig and 11 pigs/pen. Pigs were fed ad libitum with the same commercial diet until they were slaughtered at 210 d (Table 1). At 180 d of age, between 08:00 and 10:00 h, a blood sample was taken after a long fastening period (24 h) from all pigs from the jugular vein using a 10 mL tube containing 18 mg of Ethylenediaminetetraacetic acid. Immediately after collection, the plasma was separated by centrifugation (3000× *g* for 10 min at 4 °C) and stored at −80 °C, until required for NEFA determination. The day before slaughter, pigs were allocated randomly to a group subjected to either a short (12 h) or a long (24 h) fasting. At slaughter, a second blood sample was obtained from each animal after the assigned fasting duration. Carcass weight was recorded and the carcass lean percentage was estimated by an online ultrasound automatic scanner (AutoFOM; SFK-Technology, Herlev, Denmark) in accordance with the prediction equation approved for Spain (Decision 2012/384/UE). At 24 h post-mortem, final pH was measured in m. longissimus thoracis (LT) and in m. semimembranosus (SM).

After carcass quartering, samples of subcutaneous fat (SF), LT, and GM were collected and vacuum packaged. These samples remained frozen at −20 °C until required for analysis. Each pig was genotyped for polymorphisms at the *SCD* promoter (rs80912566) [6] and at the *LEPR* exon 15 (rs709596309) [9].

### 2.2. Fatty Acid Composition of Adipose Ttissue and Muscles

A tissue homogenate aliquot containing approximately 5 mg of lipids was used to obtain fatty acid methyl esters [10]. The esters were obtained using 2 mL of 20% boron trifluoride in methanol (Prolabo, Vaugereau, France), along with the extraction by hexane (Merck, Darmstadt, Germany). The quantification of fatty acid methyl esters was performed by gas chromatography using a capillary column DB23 (30 m × 0.25 mm × 0.25 µm; Agilent Technologies, Santa Clara, California, USA) and a flame ionization detector with hydrogen as the carrier gas. The samples were run under a constant flow of 2 mL/min. The oven temperature program was set at 150 °C for the first minute, then increased to 180 °C at 25 °C /min, and then further increased to 220 °C at 5 °C /min, and was finally maintained at 220 °C for 1 min. The injector and detector temperatures were 250 and 260 °C, respectively. The quantification of myristic acid (C14:0), palmitic acid (C16:0), palmitoleic acid (C16:1), stearic acid (C18:0), oleic acid (C18:1), linoleic acid (C18:2), linolenic acid (C18:3), arachidic acid (C20:0), (11Z, 14Z)-eicosadienoic acid (C20:2), and arachidonic acid (C20:4) was carried out in duplicate through area normalization after the addition of 1,2,3-tripentadecanoylglycerol (Sigma–Aldrich, Madrid, Spain) into each sample as an internal standard. Fatty acids were identified by comparing their relative retention times with those of the external standard 37-component fatty acid methyl ester mix (Sigma–Aldrich). Intramuscular fat (IMF) content was calculated as the sum of each individual fatty acid expressed as triglyceride equivalents. [11] The NEFA profile has been calculated in two ways: in absolute value and expressed as ug NEFA/mL of plasma or as the percentage of each individual NEFA relative to total NEFA.

### 2.3. Circulating NEFA Determination

The circulating NEFA extraction was performed as previously described [8] with some modifications. Briefly, 20 µL of plasma was mixed with 200 µL of C16:0-d31 (Sigma–Aldrich) in isopropanol (Prolabo) at 250 ng/mL. This was stirred for 30 min and then centrifuged for 10 min at 2,300× *g* and 20 °C. From the supernatant, 50 µL was collected and filtered through a 0.20 µm hydrophilic polytetrafluoroethylene membrane prior to injection. An Ultra-High-Performance Liquid Chromatography system coupled to a XevoTQS mass spectrometer (Waters, Milford, MA, USA) was used for the analysis. The system was equipped with an electrospray ionization source and an ACQUITY UPLC column, HSS –T3 (2.1 × 150 mm; 1.8 µm particle size). The injection volume was kept constant at 2.5 µL. Equilibration was performed for 1 min with 25% of eluent A, Milli-Q water (Millipore, Milford, USA) containing 10 mM ammonium formiate (Sigma–Aldrich), 0.15% formic acid (Sigma–Aldrich) and 75 % of eluent B (acetonitrile/isopropanol (Prolabo), 80/20 vol/vol) at 500 µL/min. At 4 min, eluent B was linearly increased to 100% and the flow rate was increased to 700 µL/min. The electrospray ionisation (ESI) source was operated under a negative mode. The operating conditions were set as follows: capillary voltage 2.5 kV, source temperature 120 °C, desolvation temperature 500 °C, cone gas flow 150 L/h, desolvation gas flow 1000 L/h, collision gas flow 0.14 mL/min, nebulizer gas flow 6.5 bar. The quantification of 12 circulating NEFA was performed using the UPLC-MRM method (Supplementary Table S1). Cone voltage was optimized individually for each fatty acid. Since the fatty acid fragmentation in electrospray ionization tandem mass spectrometry ESI/MS/MS is very poor, pseudo molecular transitions were used [8]. For each fatty acid, Q1 and Q3 mass filters were set to the monoisotopic [M-H]^–1^ mass at two collision energy levels 2 and 20 eV. The ratio of both transitions was used for confirmation and the highest signal was used for the quantification purposes. Calibration curves were created using commercially available fatty acids that were diluted to a series of appropriate concentrations using delipidated porcine plasma. Plasma delipidation was performed as described previously [12]. The data were processed using the QuanLynx^®^ V4.1 software (Waters, Milford, MA, USA), with C16:0 fatty acid-d31 as internal standard.

### 2.4. Statistical Analysis

The NEFA were analyzed using a mixed model. The model included the animal as a random effect and the batch (2 levels), the cross of age at blood extraction with fasting duration (180 d/long, 210 d/short, or 210 d/long), the *SCD* genotype (TT, CT, or CC) and the *LEPR* genotype (TT, CT, or CC) as fixed effects. Fatty acids at 210 d of age were compared among tissues using a similar model but replacing the cross of age and fasting duration with tissue (SF, LT, and GM) and fasting duration (short or long). Desaturation indices monounsaturated fatty acids/saturated fatty acids (MUFA/SFA), C18:1/C18:0 and C16:1/C16:0 were also analyzed as indicators of *SCD* activity. Fixed effects were tested using an F-test. Multiple pairwise comparisons were tested using Tukey’s HSD test. Meat traits were regressed on circulating NEFA, fasting duration (short or long), and age at blood extraction (180 or 210 d). Training (70%) and validation (30%) sets were randomly separated from the available data. All analyses were performed using JMP 12.0.1 statistical software (SAS Institute Inc., Cary, NC, USA).

## 3. Results

### 3.1. Circulating NEFA Composition

**Figure 1:**
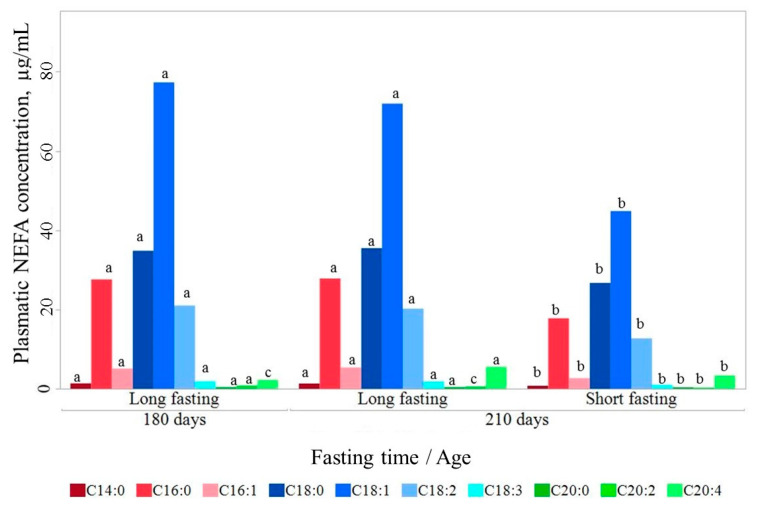
Circulating non-esterified fatty acids (NEFA) content at 180 and 210 days of life. Fatty acids lacking a common letter between age by fasting duration class differ significantly (*p* < 0.05).

In the life span between 180 and 210 days, absolute circulating NEFA content remained stable after a long fasting (172.69 ± 4.27 μg/mL and 170.78 ± 6.35 μg/mL, respectively; Figure 1). Only small changes in C20:2 and C20:4 were found, with C20:2 content being higher at 180 d than at 210 d of age (0.80 ± 0.03 vs 0.43 ± 0.03 μg/mL; *p* < 0.05) and C20:4 lower (2.26 ± 0.15 vs 4.39 ± 0.16 μg/mL; *p* < 0.05).

In contrast, circulating NEFA changed substantially with fasting duration. The total circulating NEFA content increased after a long fasting. At 210 d of age, the total circulating NEFA content was 1.5 higher in long-fasted (170.78 ± 3.99 μg/mL) than in short-fasted pigs (110.57 ± 4.08 μg/mL; *p* < 0.001). This increase happened consistently for all individual fatty acids, except for C20:2, but unevenly, thereby also modifying the relative composition. Thus, long fasting resulted in a relative composition with proportionally more monounsaturated fatty acids (MUFA) and less saturated fatty acids (SFA), and similar polyunsaturated fatty acids (PUFA) (Table 2).

The MUFA/SFA ratio, as well as the C18:1/C18:0 and C16:1/C16:0 ratios, increased after a long fasting. Compared to SF, LT, and GM, the circulating NEFA composition was characterized by a lower percentage of MUFA (Table 2), percentage of PUFA similar to SF and higher than LT and GM, and percentage of SFA around that of LT and GM, and greater than that of SF. Circulating NEFA also had a different composition within SFA, where the percentages of medium-chain fatty acids (C14:0 and C16:0) were halved and those of longer-chain fatty acids (C18:0 and C20:0) doubled relative to the other tissues. Within MUFA, differences between plasma NEFA and other tissues can be mostly attributed to C18:1 and, to a far lesser extent, to C16:1. For PUFA, the most remarkable change was for C20:4, with NEFA containing almost twice the amount in muscle and almost tenfold that in SF. The percentages of C18:2 and C18:3 were greater in NEFA than in muscle and more similar to SF, particularly C18:3.

### 3.2. Effect of SCD and LEPR on Circulating NEFA Composition

The *SCD* and *LEPR* polymorphisms had the expected effects on meat quality traits. (Supplementary Table S2). Their effects on circulating NEFA after fasting are given in Table 3.

The *SCD* genotype did not affect circulating NEFA, including the MUFA/SFA, C18:1/C18:0 and C16:1/C16:0 ratios. In contrast, the *LEPR* genotype had a marked effect on the total amount of circulating NEFA. Despite being fatter than the other two genotypes (Supplemental Table S2), TT pigs had ~18% lower total circulating NEFA than CC and CT pigs, which did not differ between them. The TT genotype decreased the circulating amount of C14:0, C16:0, C16:1, C18:1, C18:2, C18:3, and C20:2 with respect to CC and CT genotypes. The effect of the TT genotype on individual fatty acids also translated into lower total content of SFA, MUFA, and PUFA. However, the TT genotype did not affect the relative fatty acid composition of NEFA.

### 3.3. Prediction of Meat Quality Traits

**Figure 2:**
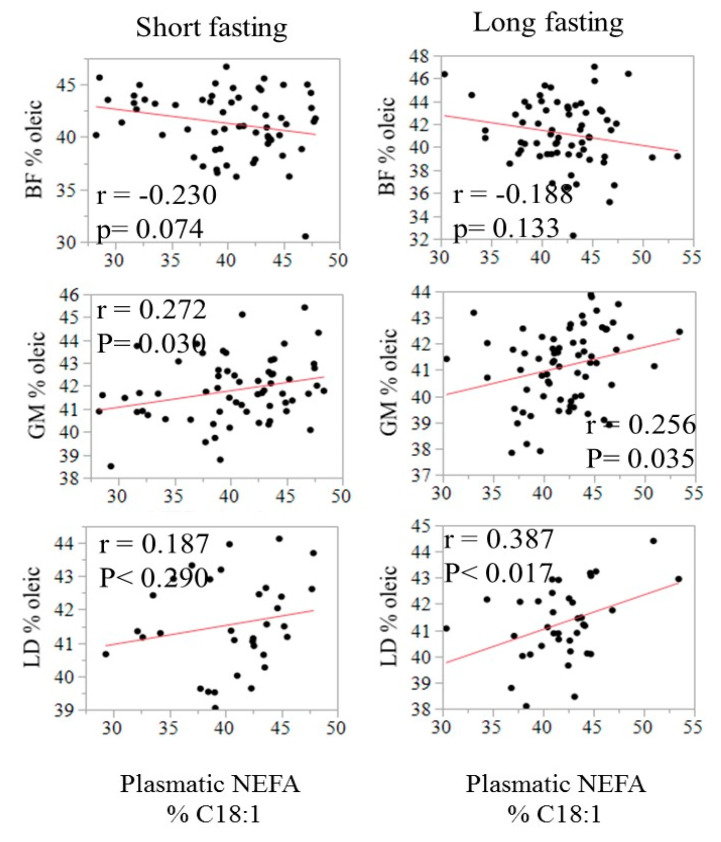
Relationship of the relative content (% of total fatty acids) of oleic acid in plasma with the relative content of oleic acid in SF, GM, and LT by fasting duration.

**Figure 3:**
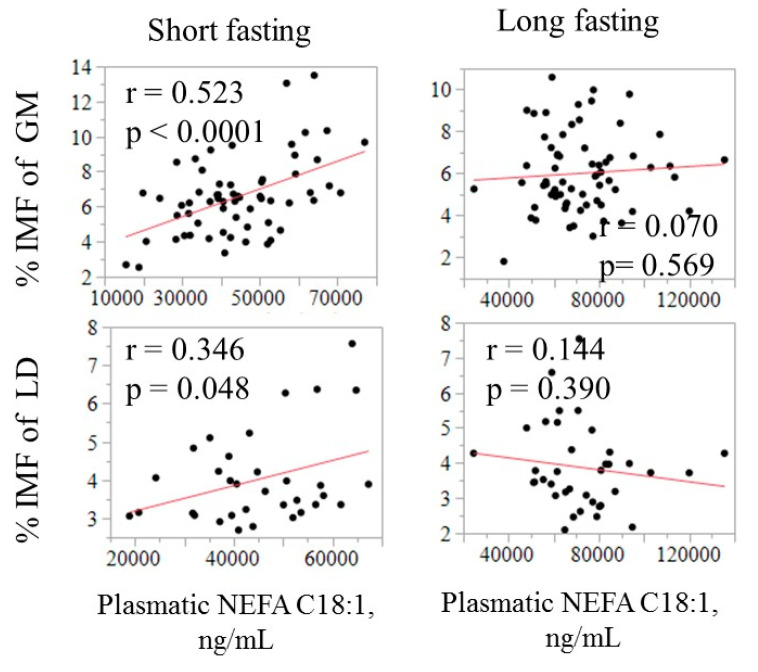
Relationship of the absolute amount of oleic acid in plasma (ng/mL) with intramuscular fat (IMF) in GM and LT by fasting duration.

The relationship between the fatty acid composition in circulating NEFA and in adipose tissues is illustrated in Figure 2 using C18:1 as an example. The correlation was negative in SF (−0.23, *p* = 0.07) but positive in both GM and LT, especially after a long fasting (0.26 to 0.39, *p* < 0.05). The relationship of C18:1 in NEFA with IMF content in LT and GM is displayed in Figure 3.

The IMF was found to be positively correlated with absolute amount of C18:1 in plasma, only after a short fasting, and was higher in GM (0.52, *p* < 0.05) than in LT (0.35, *p* < 0.05). The ability of the NEFA composition to predict some meat quality-related traits was evaluated by using all available fatty acids in a multiple regression model. The model that included the whole circulating NEFA profile as independent variables only explained a small part of the variation in IMF and C18:1 in muscle (R^2^ ranging from 0.17 to 0.29; Table 4).

## 4. Discussion

Circulating NEFA presented a singular fatty acid composition that differs from other fat depots such as SF or muscle. Despite variations caused by fasting duration, a specific pattern in circulating NEFA composition could be discerned, characterized by a high percentage of C18:0 and C20:4 relative to adipose tissue and muscle. Similar features have already been described in humans [4]. The high percentage of these two fatty acids in circulating NEFA cannot be explained by fat composition in the diet, which only contains 6.6% of C18:0 and 0.3% of C20:4 (Table 1).

The circulating NEFA content were more influenced by fasting duration than by age. Circulating NEFA are released into the bloodstream from two major sources: (a) by lipase-mediated hydrolysis of adipose tissue [13], and (b) by the action of lipoprotein lipase on dietary chylomicrons [14]. Thus, the impact of meals and ingestion patterns on circulating NEFA content may lead to the development of a circulating NEFA circadian rhythm. In humans, circulating NEFA levels increase gradually after an overnight fasting and fall dramatically in the postprandial state [15]. To the best of our knowledge, there is no study reported in fattening pigs regarding the circadian rhythm of NEFA, but our results are consistent with this pattern. Our results showed that NEFA increase with fasting duration and evidenced that, even in favorable energy balance states, NEFA content after fasting can reflect catabolic activity in the adipose tissue [16].

The physiological regulation of lipids in the bloodstream is dependent upon hormonal signals that direct both esterification and lipolysis [17]. The most well-known examples of signaling hormones are catecholamines and insulin, but there are several other signaling molecules, such as growth hormones or cytokines, which are also capable of influencing this process [18]. Fat mobilization has been described as a selective release of individual fatty acids from the adipose tissue, which is dependent on both chain length and number of double bonds. This could explain the high concentration of C20:4 in circulating NEFA, which together with C20:5 have the highest mobilization rate into plasma [19,20,21]. However, this does not explain the high levels of C18:0 in NEFA, which is not easily released from adipose stores. Therefore, the high level of C18:0 in NEFA could be attributed to its low clearance from blood. It is well-established that plasma clearance rates for NEFA varies for different fatty acids. In humans, it has been found that the clearance rate for C18:0 in blood was 30% lower than for C16:0, and that levels of C18:0 were higher in NEFA than in adipose tissue, especially under lipolysis inhibition [22]. As compared to fattening pigs, the NEFA composition in pre-farrowing, farrowing and lactating sows, which show a negative energy balance, is much poorer in C18:1 (4 to 10-times), C18:2 (10-times), and C20:4 (below detection limit) [23].

The favorable effect of the allele T of the *SCD* polymorphism (rs80912566) on fat desaturation [6] could not be verified in NEFA, possibly because *SCD* activity is involved in the anabolic process of adipose tissues whereas circulating NEFA are mainly from the diet and only less than 10% of total plasma acyl chains are from the catabolism of adipose tissue [4]. The *SCD* effect on blood lipid composition could be therefore more noticeable if the analysis was restricted to very low-density lipoproteins as an output of hepatic lipogenesis. The enhancer effect of TT pigs for the *LEPR* gene (rs709596309) on fat content [7] impacts the NEFA content but not its composition. The fact that the TT pigs were fatter and presented the lowest amount of NEFA in blood suggests that these animals have less intense lipid catabolism [7]. This result agrees with previous findings reporting higher circulating NEFA content in leaner pig breeds [24], but it contrast with reports of a moderate but significant positive association between NEFA content and body mass index in humans [25].

This information, besides being physiologically relevant, may be associated with pork quality, since muscle and fat characteristics are largely determined by the physiological status of the pig. We investigated the possible role of circulating NEFA as biomarkers for IMF content and fatty acid composition. Duroc pigs are often used for premium dry-cured products, where IMF and C18:1 are key quality features that cannot be easily measured directly in the breeding animals. Unlike other characteristics such as backfat thickness that can be accurately measured by ultrasound, measuring IMF and C18:1 content of meat in vivo is technically difficult and costly. In pig improvement programs dedicated to high quality products, being able to estimate these variables with precision on alive selection candidates would be of great interest. Our results indicate that C18:1 in circulating NEFA after a short fasting is moderately correlated with IMF and C18:1 content in muscle, in line with previous findings in humans [26,27], but that the correlation is much lower than reported for exogenous fatty acids, such as odd-chain and omega-3 fatty acids, which are commonly used as markers for specific fat sources. Moreover, the magnitude of this correlation, particularly with IMF, depended on fasting duration (Figure 3). This highlights that circulating NEFA are not very sensitive in detecting changes in blood resulting from fatty acid mobilization in IMF. An explanation for this is that C18:1 is only moderately released from adipose tissue [19] and that IMF is quantitatively a marginal storage tissue. In humans, the circulating NEFA composition of the postprandial state has been proposed as a surrogate marker for subcutaneous adipose tissue, and indirectly of diet [4,27]. Similarly to humans [28], the predictive capacity of IMF and C18:1 did not improve even when the whole circulating NEFA composition is used in a multiple regression approach.

The multiple regression approach was used to assess the predictive capacity of NEFA for carcass lean percentage and meat quality traits. In general, predictive capacity was very low except for pH in SM. Muscle pH at 24 h in SM was explained by the fasting duration and the circulating level of C14:0. The final pH reflects the way in which muscular fibers metabolize the last available energy reserves. In such a scenario, glycogen reserves are influenced by fasting duration. The positive effect of circulating C14:0 on pH (R^2^ of 0.39; *p* < 0.001) suggests that a high level of C14:0 in the bloodstream is indicative of low energy reserve in the muscular fibers.

## 5. Conclusions

The composition of circulating NEFA differs from that of adipose tissue and muscles and reflects the metabolic status of an animal at a given time. Circulating NEFA content does not show significant changes between 180 and 210 days of life. In contrast, fasting duration notably increases their content in blood. TT pigs for the *LEPR* polymorphism had a clearly lower level of circulating NEFA, but the polymorphism in the *SCD* gene had no effect. The circulating NEFA profile had a limited value as biomarkers of IMF content and fatty acid composition.

## Figures and Tables

**Table 1:** Nutrient content of the diet fed during the finishing period (as-fed basis).

Item (Feed Basis)	Experimental Diet
Net energy, kcal/kg	2400
Nutrient content, g/kg	-
Dry matter	886
Crude protein	142
Sum of EAA *	60.4
Crude fibre	36
Ether extract	64
Fatty acids, ‰ on total fatty acid basis	-
C12:0	1.1
C14:0	10.1
C16:0	20.4
C18:0	66.2
C20:0	2.0
C22:0	0.7
SFA ^†^	100.5
C14:1,cis-9	199.8
C16:1,cis-9	2.4
C18:1,cis-9	346.3
C18:1,cis11	29.8
C20:1,cis-11	8.9
C22:1,cis-13	0.5
MUFA ^‡^	587.7
C18:2,cis-9,12	273.8
C18:3,cis-9,12,15	28.5
C20:2,cis-11,14	5.4
C20:3,cis-11,14,17	1.0
C20:4,cis-5,8,11,14	3.1
PUFA ^§^	311.8

* EAA = sum of essential amino acids (Lysine, Methionine, Threonine, Isoleucine, Valine, Phenylalanine, Leucine and Histidine). ^†^ SFA: C12:0 + C14:0 + C16:0 + C18:0 + C20:0 + C22:0. ^‡^ MUFA: C14:1 cis-9 + C16:1 cis-9 + C18:1,cis-11 + C18:1,cis-9 + C20:1,cis-11 + C22:1,cis-13. PUFA ^§^: C18:2,cis-9,12 + C18:3,cis-9,12,15 + C20:2,cis-11,14 + C20:3,cis-11,14,17 + C20:4,cis-5,8,11,14.

**Table 2:** Circulating NEFA after 12-h (short) and 24-h (long) fasting and fatty acid composition of tissues.

Fatty Acid (%) ^†^	Circulating NEFA		SF	LT	GM
	Short Fasting	Long Fasting			
C14:0	0.71 ± 0.02 ^b^	0.78 ± 0.02 ^b^	1.51 ± 0.01 ^a^	1.52 ± 0.02 ^a^	1.57 ± 0.01 ^a^
C16:0	16.06 ± 0.18 ^c^	16.24 ± 0.17 ^c^	21.76 ± 0.12 ^b^	25.44 ± 0.16 ^a^	25.53 ± 0.12 ^a^
C16:1	2.26 ± 0.08 ^d^	3.03 ± 0.08 ^c^	2.34 ± 0.05 ^d^	3.69 ± 0.07 ^a^	3.39 ± 0.05 ^b^
C18:0	24.13 ± 0.26 ^a^	21.03 ± 0.26 ^b^	10.28 ± 0.28 ^d^	12.79 ± 0.24 ^c^	12.58 ± 0.17 ^c^
C18:1	40.13 ± 0.31 ^c^	42.21 ± 0.31 ^d^	46.69 ± 0.21 ^a^	45.74 ± 0.28 ^ab^	45.40 ± 0.21 ^b^
C18:2	11.79 ± 0.25 ^b^	11.65 ± 0.24 ^b^	14.14 ± 0.17 ^a^	7.22 ± 0.23 ^d^	8.36 ± 0.16 ^c^
C18:3	0.92 ± 0.02 ^b^	1.06 ± 0.02 ^a^	1.02 ± 0.01 ^a^	0.34 ± 0.01 ^d^	0.46 ± 0.01 ^c^
C20:0	0.34 ± 0.006 ^a^	0.26 ± 0.006 ^b^	0.15 ± 0.004 ^d^	0.19 ± 0.005 ^c^	0.15 ± 0.004 ^d^
C20:1	-	-	0.99 ± 0.008 ^a^	0.73 ± 0.01 ^b^	0.73 ± 0.01 ^b^
C20:2	0.27 ± 0.01 ^d^	0.35 ± 0.01 ^c^	0.77 ± 0.007 ^a^	0.33 ± 0.01 ^c^	0.39 ± 0.007 ^b^
C20:4	3.27 ± 0.14 ^a^	3.27 ± 0.14 ^a^	0.32 ± 0.10 ^d^	1.96 ± 0.13 ^b^	1.39 ± 0.10 ^c^
SFA *	41.27 ± 0.35 ^a^	38.30 ± 0.34 ^c^	33.72 ± 0.24 ^d^	39.92 ± 0.32 ^b^	39.84 ± 0.24 ^b^
MUFA ^†^	42.40 ± 0.36 ^c^	45.24 ± 0.35 ^b^	50.02 ± 0.24 ^a^	49.92 ± 0.32 ^a^	49.51 ± 0.24 ^a^
PUFA ^‡^	16.24 ± 0.38 ^a^	16.35 ± 0.38 ^a^	16.26 ± 0.26 ^a^	9.87 ± 0.35 ^b^	10.61 ± 0.25 ^b^
C16:1/C16:0	0.13 ± 0.005 ^b^	0.19 ± 0.005 ^a^	0.10 ± 0.003 ^c^	0.14 ± 0.004 ^b^	0.13 ± 0.003 ^b^
C18:1/C18:0	1.71 ± 0.05 ^d^	2.07 ± 0.05 ^c^	4.30 ± 0.03 ^a^	3.27 ± 0.05 ^b^	3.31 ± 0.03 ^b^
MUFA/SFA	1.04 ± 0.01 ^c^	1.19 ± 0.01 ^b^	1.49 ± 0.01 ^a^	1.25 ± 0.01 ^b^	1.24 ± 0.01 ^b^

^†^ Least-square means ± standard error; * SFA: C14:0 + C16:0 + C18:0 + C20:0. MUFA: C16:1 + C18:1 + C20:1. ^‡^ PUFA: C18:2 + C18:3 + C20:2 + C20:4; SF: subcutaneous fat; LT: muscle longissimus thoracis; GM: muscle gluteus medius. ^a–d^ Within row, means with different superscripts differ significantly (*p* ≤ 0.05).

**Table 3:** Absolute levels of circulating non-esterified fatty acids by *SCD* and *LEPR* genotype.

NEFA (µg/mL) ^†^	*SCD*			*LEPR*		
	CC	CT	TT	CC	CT	TT
C14:0	1.19 ± 0.06	1.20 ± 0.05	1.12 ± 0.08	1.23 ± 0.05 ^a^	1.30 ± 0.06 ^a^	0.98 ± 0.09 ^b^
C16:0	24.87 ± 0.93	24.71 ± 0.78	23.81 ± 1.20	25.05 ± 0.76 ^a^	27.18 ± 0.88 ^a^	21.15 ± 1.33 ^b^
C16:1	4.25 ± 0.28	4.44 ± 0.24	4.64 ± 0.36	4.93 ± 0.23 ^a^	4.90 ± 0.26 ^a^	3.50 ± 0.40 ^b^
C18:0	32.90 ± 0.84	32.83 ± 0.71	31.25 ± 1.08	32.76 ± 0.68 ^a^	34.81 ± 0.79 ^a^	29.40 ± 1.20 ^a^
C18:1	67.62 ± 2.25	66.32 ± 7.90	66.38 ± 2.91	68.65 ± 1.84 ^a^	72.89 ± 2.14 ^a^	58.78 ± 3.23 ^b^
C18:2	18.12 ± 0.84	18.12 ± 0.71	17.65 ± 1.09	18.76 ± 0.69 ^a^	20.12 ± 0.79 ^a^	15.01 ± 1.20 ^b^
C18:3	1.63 ± 0.10	1.68 ± 0.08	1.60 ± 0.13	1.76 ± 0.08 ^a^	1.79 ± 0.09 ^a^	1.36 ± 0.14 ^b^
C20:0	0.42 ± 0.01	0.42 ± 0.01	0.41 ± 0.01	0.42 ± 0.01	0.42 ± 0.01	0.41 ± 0.01
C20:2	0.61 ± 0.03	0.60 ± 0.03	0.54 ± 0.04	0.61 ± 0.02 ^a^	0.70 ± 0.03 ^a^	0.45 ± 0.05 ^b^
C20:4	3.41 ± 0.20	3.27 ± 0.17	3.13 ± 0.26	3.34 ± 0.17	3.35 ± 0.19	3.11 ± 0.29
SFA *	59.38 ± 1.70	59.15 ± 1.44	56.31 ± 2.22	59.33 ± 1.0 ^a^	63.66 ± 1.62 ^a^	51.86 ± 1.45 ^b^
MUFA ^†^	72.04 ± 2.39	70.78 ± 2.02	69.16 ± 3.11	72.60 ± 1.96 ^a^	77.51 ± 2.27 ^a^	61.88 ± 3.43 ^b^
PUFA ^‡^	23.77 ± 1.07	23.68 ± 0.90	22.92 ± 1.39	24.48 ± 0.88 ^a^	25.97 ± 1.01 ^a^	19.92 ± 1.54 ^b^
C16:1/C16:0	0.16 ± 0.005	0.17 ± 0.005	0.17 ± 0.007	0.18 ± 0.004 ^a^	0.17 ± 0.005 ^a^	0.15 ± 0.008 ^b^
C18:1/C18:0	2.04 ± 0.046	2.03 ± 0.039	2.12 ± 0.060	2.08 ± 0.037	2.09 ± 0.044	2.03 ± 0.066
MUFA/SFA	1.19 ± 0.024	1.19 ± 0.020	1.23 ± 0.031	1.21 ± 0.020	1.12 ± 0.023	1.18 ± 0.035
TOTAL	154 ± 4.95	153 ± 4.18	151 ± 6.4	158 ± 4.05 ^a^	167 ± 4.69 ^a^	134 ± 7.12 ^b^

^†^ Least-square means ± SE; * SFA: C14:0 + C16:0 + C18:0 + C20:0. †MUFA: C16:1 + C18:1 + C20:1. ^‡^ PUFA: C18:2 + C18:3 + C20:2 + C20:4; ^a,b^ Within row and genotype, means with different superscripts differ significantly (*p* ≤ 0.05).

**Table 4:** Prediction of carcass and meat quality traits using quantitative NEFA composition.

Trait		Training Set		Validation Set		Significant Model Effects (*p* ≤ 0.05) ^†^
		n	R^2^	N	R^2^	
Carcass Lean, %		191	0.152	94	0.016	NEFA C18:1
pH LT *		188	0.306	93	0.117	No significant effects
pH SM *		188	0.458	93	0.360	Fasting duration, NEFA C14:0
IMF, %	LT	101	0.176	50	−0.091	NEFA C14:0, C18:2, C20:0
	GM	186	0.172	91	0.037	NEFA C18:2, C20:0
C18:1, % ^‡^	SF	181	0.273	90	0.235	Fasting duration, NEFA C14:0, C18:0, C18:1, C20:0
	LT	101	0.212	48	0.154	No significant effects
	GM	188	0.292	91	0.078	Age, Fasting duration
MUFA/SFA	SF	181	0.334	90	0.038	Age, Fasting duration, NEFA C14:0, C16:1
	LT	101	0.156	48	−0.005	NEFA C18:3
	GM	177	0.260	90	0.142	Age, Fasting duration, NEFA C14:0, C16:1

* pH measured at 24 h post mortem. ^†^ The set of independent variables are NEFA content (ng/mL), fasting duration (short or long) and age. ^‡^ On total fatty acid basis. IMF: intramuscular fat; C18:1: oleic acid content; MUFA: monounsaturated fatty acids; SFA: saturated fatty acids; SF: subcutaneous fat; LT: m. longissimus thoracis; GM: m. gluteus medius; SM: m. semimembranosus.

## Data Availability

The data presented in this study are available on request from the corresponding author.

## Supplementary Materials

The following are available online at https://www.mdpi.com/2076-2615/11/2/386/s1, Table S1: Precursor and product Ion pairs and parameters for Multiple Reaction Monitoring of quantified fatty acids and the internal standard, Table S2: Least-square means (±SE) for carcass and meat quality traits by *SCD* and *LEPR* genotype.

## References

[1] Wood J.D., Enser M., Fisher A.V., Nute G.R., Sheard P.R., Richardson R.I., Hughes S.I., Whittington F.M. (2008). Fat deposition, fatty acid composition and meat quality: A review. Meat Sci..

[2] Summers L.K.M., Fielding B.A., Herd S.L., Ilic V., Clarck M.L., Quinlan P.T., Frayn K.N. (1999). Use of structured triacylglycerols containing predominantly stearic and oleic acids to probe early events in metabolic processing of dietary fat. J. Lipid Res..

[3] Jelic K., Hallgreen C.E., Colding-Jorgensen M. (2009). A Model of NEFA Dynamics with Focus on the Postprandial State. Ann. Biomed. Eng..

[4] Hodson L., Skeaff C.M., Fielding B.A. (2008). Fatty acid composition of adipose tissue and blood in humans and its use as a biomarker of dietary intake. Prog. Lipid Res..

[5] O’Hea E.K., Leveille G.A. (1969). Significance of Adipose Tissue and Liver as Sites of Fatty Acid Synthesis in the Pig and the Efficiency of Utilization of Various Substrates for Lipogenesis. J. Nutr..

[6] Estany J., Ros-Freixedes R., Tor M., Pena R.N. (2014). A Functional Variant in the Stearoyl-CoA Desaturase Gene Promoter Enhances Fatty Acid Desaturation in Pork. PLoS ONE.

[7] Ros-Freixedes R., Gol S., Pena R.N., Tor M., Ibanez-Escriche N., Dekkers J.C.M., Estany J. (2016). Genome-Wide Association Study Singles Out *SCD* and *LEPR* as the Two Main Loci Influencing Intramuscular Fat Content and Fatty Acid Composition in Duroc Pigs. PLoS ONE.

[8] Hellmuth C., Weber M., Koletzko B., Peissner W. (2012). Nonesterified Fatty Acid Determination for Functional Lipidomics: Comprehensive Ultrahigh Performance Liquid Chromatography-Tandem Mass Spectrometry Quantitation, Qualification, and Parameter Prediction. Anal. Chem..

[9] Ovilo C., Fernández A., Noguera J.L., Barragan C., Leton R., Rodríguez C., Mercadé A., Álves E., Folch J.M., Varona L. (2005). Fine mapping of porcine chromosome 6 QTL and *LEPR* effects on body composition in multiple generations of an Iberian by Landrace intercross. Genet. Res..

[10] Rule D.C. (1997). Direct transesterification of total fatty acids of adipose tissue, and of freeze-dried muscle and liver with boron-trifluoride in methanol. Meat Sci..

[11] AOAC (Association of Analytical Chemists) (2000). Official Method 960.39 Fat (Crude) or Ether Extract in Meat. Official Methods of Analysis.

[12] Cham B.E., Knowles B.R. (1976). 1976. Solvent system for delipidation of plasma or serum without protein precipitation. J. Lipid Res..

[13] Coppack S.W., Evans R.D., Fisher R.M., Frayn K.N., Gibbons G.F., Humphreys S.M., Kirk M.L., Potts J.L., Hockaday T.D.R. (1992). Adipose-tissue metabolism in obesity: Lipase action in vivo before and after a mixed meal. Metab. Clin. Exp..

[14] Fielding B.A., Callow J., Owen R.M., Samra J.S., Matthews D.R., Frayn K.N. (1996). Postprandial lipemia: The origin of an early peak studied by specific dietary fatty acid intake during sequential meals. Am. J. Clin. Nutr..

[15] Frayn K.N., Williams C.M., Arner P. (1996). Are increased plasma nonesterified fatty acid concentrations a risk marker for coronary heart disease and other chronic diseases?. Clin. Sci..

[16] Frayn K.N., Shadid S., Hamlani R., Humphreys S.M., Clark M.L., Fielding B.A., Boland O., Coppack S.W. (1994). Regulation of fatty-acid movement in human adipose-tissue in the postabsorptive-to-postprandial transition. Am. J. Physiol..

[17] Stich V., Berlan M. (2004). Physiological regulation of NEFA availability: Lipolysis pathway. Proc. Nutr. Soc..

[18] Lafontan M., Langin D. (2009). Lipolysis and lipid mobilization in human adipose tissue. Prog. Lipid Res..

[19] Connor W.E., Lin D.S., Colvis C. (1996). Differential mobilization of fatty acids from adipose tissue. J. Lipid Res..

[20] Halliwell K.J., Fielding B.A., Samra J.S., Humphreys S.M., Frayn K.N. (1996). Release of individual fatty acids from human adipose tissue in vivo after an overnight fast. J. Lipid Res..

[21] Raclot T. (2003). Selective mobilization of fatty acids from adipose tissue triacylglycerols. Prog. Lipid Res..

[22] Mittendorfer B., Liem O., Patterson B.W., Miles J.M., Klein S. (2003). What does the measurement of whole-body fatty acid rate of appearance in plasma by using a fatty acid tracer really mean?. Diabetes.

[23] Contreras G.A., Kirkwood R.N., Sordillo L.M. (2013). Mononuclear leukocyte fatty acid composition and inflamatory phenotype in periparturient and lactating sows. J. Anim. Sci..

[24] Yang B., Bassols A., Saco Y., Perez-Enciso M. (2011). Association between plasma metabolites and gene expression profiles in five porcine endocrine tissues. Genet. Sel. Evol..

[25] Il’yasova D., Wang F., D’Agostino R.B., Hanley A., Wagenknecht L.E. (2010). Prospective association between fasting NEFA and type 2 diabetes: Impact of post-load glucose. Diabetologia.

[26] Yli-Jama P., Haugen T.S., Rebnord H.M. (2001). Selective mobilization of fatty acids from human adipose tissue. Eur. J. Intern. Med..

[27] Hellmuth C., Demmelmair H., Schmitt I., Peissner W., Bluher M., Koletzko B. (2013). Association between Plasma Nonesterified Fatty Acids Species and Adipose Tissue Fatty Acid Composition. PLoS ONE.

[28] Walker C.G., Browning L.M., Stecher L., West A.L., Madden J., Jebb J.A., Calder P.C. (2015). Fatty acid profile of plasma NEFA does not reflect adipose tissue fatty acid profile. Br. J. Nutr..

