# Supplementary material for: Circulating Non-Esterified Fatty Acids as Biomarkers for Fat Content and Composition in Pigs
Source: Animals (Basel). 2021 Feb 3;11(2):386. doi: 10.3390/ani11020386 (PMC7913534; doi:10.3390/ani11020386)
Supplement: Supplementary file 1 [file animals-11-00386-s001.pdf]

**Supplemental Table S1.** Precursor/Product Ion pairs and parameters for Multiple Reaction Monitoring of quantified fatty acids and the internal standard.

| Fatty acid | Retention time (min) | $[M-H]^-$ (*m/z*) | MRM Transition | Cone voltage (V) | Collision energy (eV) |
|---|---|---|---|---|---|
| C14:0 | 1.83 | 227.18 | 227.18 > 227.18 | 2.0 | 2.0 |
| | | | 227.18 > 227.18 | 2.0 | 20.0 |
| C15:0 | 2.09 | 241.26 | 241.26 > 241.26 | 4.0 | 2.0 |
| | | | 241.26 > 241.26 | 4.0 | 20.0 |
| C16:0 | 2.36 | 255.27 | 255.27 > 255.27 | 2.0 | 2.0 |
| | | | 255.27 > 255.27 | 2.0 | 20.0 |
| C16:1 | 1.91 | 253.26 | 253.26 > 253.26 | 60.0 | 2.0 |
| | | | 253.26 > 253.26 | 60.0 | 20.0 |
| C16:0-d31 | 1.91 | 286.27 | 286.27 > 286.27 | 2.0 | 2.0 |
| | | | 286.27 > 286.27 | 2.0 | 20.0 |
| C18:0 | 2.90 | 283.30 | 283.30 > 283.30 | 72.0 | 2.0 |
| | | | 283.30 > 283.30 | 72.0 | 20.0 |
| C18:1 | 2.43 | 281.35 | 281.35 > 281.35 | 32.0 | 2.0 |
| | | | 281.35 > 281.35 | 32.0 | 20.0 |
| C18:2 | 2.03 | 279.27 | 279.27 > 279.27 | 66.0 | 2.0 |
| | | | 279.27 > 279.27 | 66.0 | 20.0 |
| C18:3 | 1.71 | 277.60 | 277.26 > 277.66 | 66.0 | 2.0 |
| | | | 277.26 > 277.66 | 66.0 | 20.0 |
| C20:0 | 3.42 | 311.27 | 311.27 > 311.27 | 84.0 | 2.0 |
| | | | 311.27 > 311.27 | 84.0 | 20.0 |
| C20:2 | 2.51 | 307.37 | 307.37 > 307.37 | 50.0 | 2.0 |
| | | | 307.37 > 307.37 | 50.0 | 20.0 |
| C20:4 | 1.93 | 303.34 | 303.34 > 303.34 | 52.0 | 2.0 |
| | | | 303.34 > 303.34 | 52.0 | 20.0 |

**Supplemental Table S2.** Least-square means (± SE) for carcass and meat quality traits by *SCD* and *LEPR* genotype

| | | *SCD* | | | *LEPR* | | |
|---|---|---|---|---|---|---|---|
| | | CC | CT | TT | CC | CT | TT |
| Carcass weight, kg | | 101.3 ± 1.2 | 101.4 ± 1.0 | 105.4 ± 1.6 | 101.1 ± 1.0[b] | 100.7 ± 1.2 | 105.9 ± 1.8 |
| Carcass lean, % | | 40.7 ± 0.7 | 41.4 ± 0.6 | 41.5 ± 0.9 | 40.3 ± 0.6 | 41.9 ± 0.7 | 41.3 ± 1.0 |
| pH | LT | 5.9 ± 0.0 | 5.9 ± 0.0 | 5.9 ± 0.0 | 5.9 ± 0.02[b] | 5.9 ± 0.0[ab] | 6.0 ± 0.03[a] |
| | SM | 5.8 ± 0.0 | 5.8 ± 0.0 | 5.9 ± 0.0 | 5.8 ± 0.02[b] | 5.8 ± 0.0[b] | 5.9 ± 0.03[a] |
| % IMF | LT | 3.9 ± 0.2 | 4.0 ± 0.1 | 4.0 ± 0.3 | 3.9 ± 0.1[ab] | 3.5 ± 0.2[b] | 4.6 ± 0.3[a] |
| | GM | 6.3 ± 0.2 | 6.4 ± 0.2 | 6.1 ± 0.3 | 6.2 ± 0.2[b] | 5.6 ± 0.2[ab] | 7.1 ± 0.3[a] |

[a-b] Within row and gene means with different superscripts differ significantly ($P \leq 0.05$). SF, subcutaneous fat; GM, m.gluteus medius; IMF, intramuscular fat; *LEPR*, leptin receptor gene; LT, m. longissimus thoracis; *SCD*, stearoyl-CoA desaturase gene; SM, m. semimembranosus.
